# Economic Feasibility of Wireless Sensor Network-Based Service Provision in a Duopoly Setting with a Monopolist Operator

**DOI:** 10.3390/s17122727

**Published:** 2017-11-25

**Authors:** Angel Sanchis-Cano, Julián Romero, Erwin J. Sacoto-Cabrera, Luis Guijarro

**Affiliations:** 1 ITACA, Universitat Politècnica de València, Camino de Vera s/n, 46022 Valencia, Spain; jurocha@itaca.upv.es (J.R.); ersacab@doctor.upv.es (E.J.S.-C.); lguijar@upv.es (L.G.); 2 GIHP4C, Universidad Politécnica Salesiana-Sede Cuenca, Calle Vieja 12-30 y Elia Liut, 010102 Cuenca, Ecuador

**Keywords:** internet of things service provider, network economics, pricing, game theory, population games, discrete choice model, Nash equilibrium, users subscription, wireless sensor networks

## Abstract

We analyze the feasibility of providing Wireless Sensor Network-data-based services in an Internet of Things scenario from an economical point of view. The scenario has two competing service providers with their own private sensor networks, a network operator and final users. The scenario is analyzed as two games using game theory. In the first game, sensors decide to subscribe or not to the network operator to upload the collected sensing-data, based on a utility function related to the mean service time and the price charged by the operator. In the second game, users decide to subscribe or not to the sensor-data-based service of the service providers based on a Logit discrete choice model related to the quality of the data collected and the subscription price. The sinks and users subscription stages are analyzed using population games and discrete choice models, while network operator and service providers pricing stages are analyzed using optimization and Nash equilibrium concepts respectively. The model is shown feasible from an economic point of view for all the actors if there are enough interested final users and opens the possibility of developing more efficient models with different types of services.

## 1. Introduction

The Internet of Things (IoT) is a key concept in the future of the Internet with several technologies involved, possible applications and open research challenges [1,2]. The traditional usage of networks where humans are the main users is changing progressively to a things centered model [3]. It is expected a mass market adoption for IoT in the next 2–7 years [4], but nowadays there are already working IoT technologies [5,6,7,8], industrial applications [9] and projects such as [10] or [11], where WSNs are used to minimize the energy consumption of heating, ventilation, and air conditioning systems. However, there is a lack of studies analyzing the economic aspects of IoT, and particularly of sensor network-based services, such as pricing or economic viability [12,13,14]. Recent investigations have shown an interest from the industry verticals to integrate IoT and 5G technologies, nevertheless it is not clear how the different telecom players could benefit from it [15,16]. Given the huge investments required to develop the new technologies [17] it is necessary the study of new business models and their economic viability as well as the emergence of new actors in the market [18]. In order to address this problem we analyze a new business model centered in the provision of sensor-data-based services from a new point of view, where the providers are the owners of the Wireless Sensor Networks (WSNs).

Despite the small number of studies, there are some interesting contributions such as [19], which proposes a new business model for WSN-based services, where virtualization of WSN is studied. The virtualization allows the author to separate the WSN infrastructure from the services offered to final users, however the model is not studied from a mathematical perspective. Another business model is studied in [20,21], where a bundling platform acts as an intermediary, buying the data from WSNs and selling data-based services to final users, however the model does not analyze the cost of collecting and transmitting the sensors’ data nor a competition scenario. The pricing mechanisms are studied in both articles using game theory and a solution maximizing the platforms’ profit is shown to exist. Another approach based on bundling is [22], where several business models are proposed, nevertheless the work is too general and does not analyze the models in depth. The work in [23] proposes several models, where users purchase providers’ IoT data through a marketplace and analyzes several economic concepts, such as value and pricing of information. In addition, it also analyzes the competition between providers using a game theory approach, nevertheless the model does not evaluate the quality of the information and how the information is transported from providers to consumers.

Network pricing has also been studied as a congestion control tool [24] and as an efficient power control mechanism [25,26], showing promising results in both, the distribution of the system load and in the control of the energy usage. It also has been used in combination with game theory and machine learning to study the competition in access networks [27], showing an improvement in the network usage and energy consumption. Nevertheless, these works are focused in very specific aspects of WSNs service provision, and they do not analyze an end-to-end business model, which provides a global point of view of all the system, from then sensors to the final users.

### 1.1. Paper Contributions and Outline

In this paper, we propose a novel model where the IoT-SPs are the owners of the WSNs, which analyzes not only the competition between Internet of Things-Service Providers (IoT-SPs), but also models how the sensing data is obtained and the related costs, . Both IoT-SPs compete to provide WSN-data-based services to final users. The WSN-data is gathered by each IoT-SP through a Network Operator (OP).In this work, we study the feasibility of the model from a positive-profit point of view for all the actors. The model is analyzed as two games with two stages each one using game theory. The first game studies the competition between WSNs in order to upload the sensing data to their IoT-SPs. The behavior of WSNs is modeled using a delay-sensitive utility function and an equilibrium is found using population games. The second game studies the IoT-SPs price competition. The game is analyzed using backward induction and a Nash equilibrium is found. Our model has the peculiarity that both games are connected, specifically the IoT-SPs-Users game is influenced by the Sinks-OP game solution. We also provide detailed mathematical procedures and graphic representations, that demonstrate the economic viability of the model for all the actors involved if the number of potential customers is high enough and their data/price ratio requirements is bounded. In addition, it also opens the possibility of improving the OP network efficiency as well as IoT-SPs profits by offering services with different data-quality requirements.

One real-life application of the proposed model is a real-time route planning service. A car manufacturer, acting as a service provider, installs location-tracking sensors in every car in order to offer a real-time route optimization service. The real-time location data is collected through an OP. The final users decide to subscribe or not based on the quality of the proposed routes and the price charged for the service. Finally, the quality of the route service is related with both, the amount of data collected and the data accuracy, which is related with the data delay.

The rest of this paper is organized as follows: in Section 2, we describe in detail the model with the actors, the utility of each actor and the pricing scheme. In Section 3, the two games of the model are described and the subscription and pricing strategies are solved. Section 4 shows and discuss the results and Section 5 draws the conclusions of the work.

## 2. General Model

**Figure 1:**
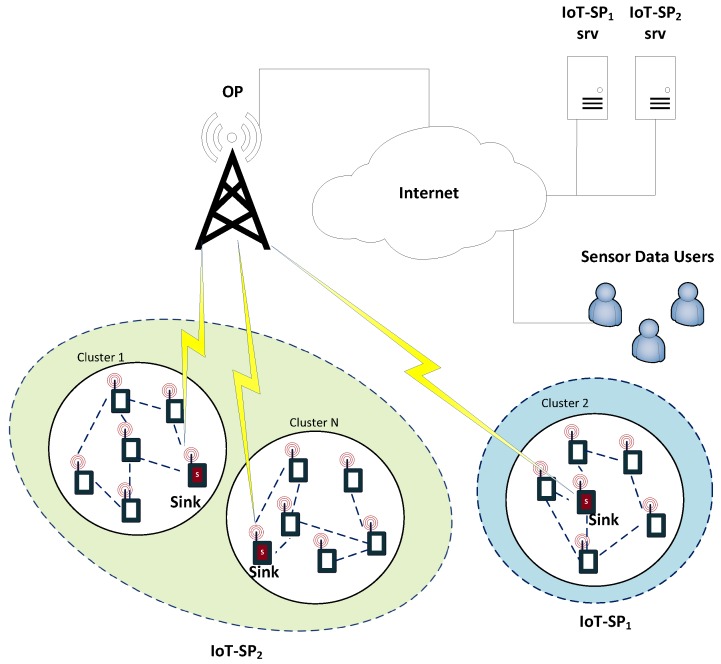
Analyzed scenario with all the actors of the market. Each IoT-SP collects its sensing data through an OP and transmits it to a server (srv) where it is processed in order to offer a service to the Sensor Data Users.

We consider the IoT scenario that is depicted in Figure 1 with two IoT-SPs deploying their private WSNs in order to provide sensor-data-based services to sensor-data users or simply final users, who pay to the IoT-SPs for this service. The sensor nodes are grouped into clusters. Each cluster has a large number of sensing nodes connected through a multi-hop wireless network [28], and belong to only one IoT-SP. Each cluster has a sink node, which transmits the data collected by all the nodes in the cluster to their IoT-SP server (IoT-SP${}_{i}$ srv) through a network operator (OP) and Internet. In the IoT-SP servers the data is aggregated in order to provide a service to final users. Our scenario has the following market actors:

- *Sinks*.
- *Network Operator* (OP).
- *Users*.
- *Internet of Things-Service Providers* (IoT-SPs).

### 2.1. Sinks

Each sink belongs to only one IoT-SP. They are responsible of transmitting all the data collected by sensors in a WSN to their IoT-SP server. They are the clients of the wireless connectivity service offered by the OP. The number of IoT-SP${}_{i}$ Sinks is $N_{j}$, where $N_{j}\gg 1$ (j=1,2), and $N_{1}+N_{2}=N$.

In order to model the utility perceived by the sinks that subscribe to the OP we use a quality function *Q* based in [29,30,31,32,33], which evaluates the service offered by the OP as a latency based service [34]:

(1) $$Q\equiv c{\left(\frac{T}{\tau }\right)}^{-1},$$

where c>0 is a conversion factor and T/τ is the mean sensing-data-unit service time normalized by the mean sensing-data-unit transmission time $\tau =\frac{1}{\mu }$, that is the minimum possible value of *T*. Note that *Q* decreases when the service time *T* increases, which means that the users perceive a worst quality when the delay of the network increases. We have chosen this function due to its ability to model the congestion in the wireless network, which makes it suitable for many IoT scenarios with delay constraints [35]. This quality function also has the ability to model different kinds of users through the value of τ and different queueing systems throught *T*, however, in this model we consider homogeneous sinks, given that we study the competition in a single service provision. We model the OP service as a M/M/1 system, and compute the mean service time *T* [36] as

(2) $$T=\frac{\tau }{1-\tau \lambda }.$$

The utility function models the perception that sinks have about the OP connectivity service. We propose a utility function for the sinks that subscribe to the OP as the difference between the quality perceived by the sinks and the price charged by the operator, also called compensated utility, which is a function widely used in economics and telecommunications [29,37,38,39,40]

(3) $$U_{s}\equiv Q-p=c\left(1-x_{1}rN\tau \right)-p,$$

where we have re-written the arrival rates as the traffic generated by all the sinks being served $\lambda =x_{1}rN$, *r* is the sensing-data-unit generation rate of one sink, *p* is the price charged by the OP to each IoT-SP${}_{j}$ (j=1,2) when its sinks transmit one sensing-data-unit and $x_{1}$ is the fraction of sinks being served by the OP. The utility must be positive $U_{s}\geq 0$, or equivalently, the price charged by the OP should not be higher than the service value perceived by the sink, otherwise the sink will not subscribe to the service. Note that all the sinks, whichever IoT-SP they belong to, perceive the same utility, which means that the fraction of sinks served by the OP is the same for all the IoT-SPs. The distribution of sinks in the system is described by the vector $X_{s}=\left(x_{0},x_{1}\right)$, where $x_{0}$ and $x_{1}$, are the fraction of sinks not being served and being served by the OP respectively and $x_{0}+x_{1}=1$.

### 2.2. Network Operator

The OP offers a wireless connectivity service to the sinks, that allows them to transmit the data collected to their IoT-SP, and charges a price *p* to the corresponding IoT-SP per sensing-data-unit transmitted.

The objective of the OP is to maximize its own profit announcing a price p>0. The OP profit is:

(4) $$\Pi_{OP}=px_{1}rN.$$

### 2.3. Users

Users want to subscribe to a sensor-data service offered by the IoT-SPs. The number of users is *M*, where M≫1. The utility of a user making the choice *j* is based on [21,41]

(5) $$U_{u_{j}}=\alpha \log\left(\frac{\beta R_{j}}{f_{j}}\right)+\kappa_{u_{j}},$$

where the first part of the expression is deterministic and is related with the market parameters while the second part $\kappa_{u_{j}}$ is treated as a random variable that models the unobserved user-specific part of the utility. The random variable $\kappa_{u_{j}}$ follows a Gumbel distribution of mean 0. The human behavior is hard to predict and usually users within the same population do not have the same preferences. For instance, while some users always prefer the cheapest option others only will change their decision if the difference in the perceived utility is high enough. All these unknown effects are aggregated in the random variable $\kappa_{u_{j}}$. In the deterministic part $R_{j}$ is the quality of the data provided by the WSN to the IoT-SP${}_{j}$, $f_{j}$ is the price per time unit that users pay to the IoT-SP${}_{j}$ for its service, β is a conversion factor and α>0 is a sensitivity parameter that models the relative importance of the rate R/f. Larger values of α increase the impact of the rate R/f in users’ choices, while lower values of α reduce the impact. In our model we set the conversion factor β=1. We obtain the expression for $R_{j}$ assuming that the quality of the information is proportional to the number of sinks sending data to the IoT-SP${}_{j}$

(6) $$R_{j}=x_{1}rN_{j}.$$

The logarithmic relation between physical magnitudes and the human perception observed in (5) has been justified in telecommunications through the Weber-Fechner Law [42,43,44].

The users will choose the IoT-SP${}_{j}$ that provides him the highest utility $U_{u_{j}}\geq U_{u_{k}}\;\forall \;k\neq j$. The distribution of users in the system is described by the vector $X_{u}=\left(y_{0},y_{1}\right)$, where $y_{0}$ is the fraction of users not subscribed to the IoT-SP and $y_{1}$ is the fraction of users subscribed to IoT-SP. Note that $y_{0}+y_{1}=1$.

### 2.4. IoT-Service Providers

The IoT-SPs are the owners of the sensors. IoT-SP${}_{j}$ pays a price *p* for each sensing-data-unit transmitted by its sinks through the OP and announces a price $f_{j}$ per time unit that will be charged to its users. According to the previous information, we can compute the IoT-SP${}_{j}$ profit as:

(7) $$\Pi_{{\mathrm{IoT-SP}}_{j}}=y_{j}Mf_{j}-x_{1}rN_{j}p=y_{j}Mf_{j}-R_{j}p,$$

where $y_{j}M$ is the number of users subscribed to the IoT-SP${}_{j}$ service and $x_{j}rN_{j}$ is the number of sensing-data-units transmitted by the sinks per time unit through the OP. The first part of the expression are the revenues obtained from the users, while the second part is the cost of transmitting the sensors data through the OP network.

**Figure 2:**
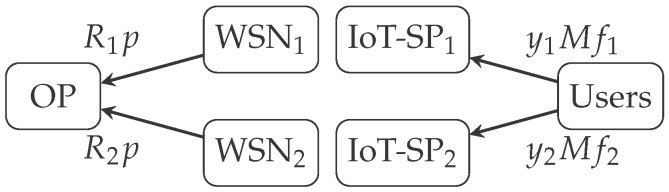
Model payments flow and actors involved.

Figure 2 shows the pricing scheme of the model described in this section, where WSN${}_{j}$ are all the sinks of the IoT-SP${}_{j}$ (j=1,2).

## 3. Game Analysis

**Figure 3:**
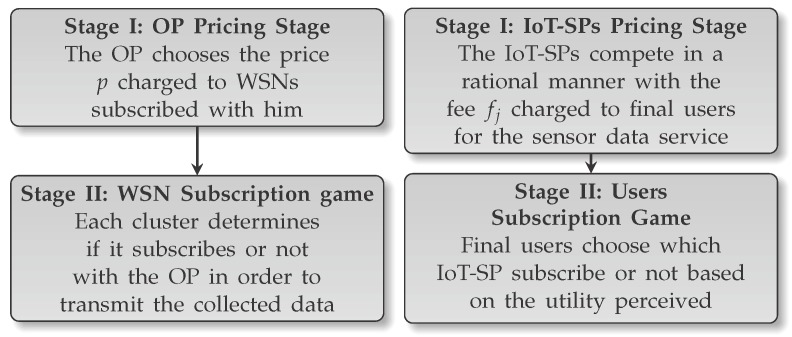
Description of the games stages.

Optimal profits could be obtained if the IoT-SPs were able to change their sinks’ decisions, however, in most real scenarios it is not possible due to energy limitations. Change sinks’ decisions implies a constant communication between the IoT-SPs and sinks, which requires a lot of energy, which typically is a limited resource in WSNs [45,46], although there are cases where the sensors could be wireless-powered [47]. In this paper, we consider the case where the energy is a limited resource, in order to be the as general as possible. Assuming that the IoT-SPs cannot influence in the decisions of their sinks, the model can be analyzed as two games of two stages. The model has the characteristic that both games are connected through the value of $R_{j}$ in (6). Both games have a similar structure: firstly a pricing stage and secondly a subscription stage. The game model is summarized in Figure 3.

The correct way forward is to solve first Game I and then solve Game II replacing the variables with the equilibrium values obtained in the solution of Game I. In the Game I, the second stage is solved using Population Games described in [48], while the pricing stage is solved using optimization methods. In the Game II, the second stage is solved using the probability of choice for the Logit model [49], while the first stage is solved using game theory and the concept of Nash Equilibrium.

Both games were solved using backward induction. Backward induction consists in deducing backwards from the end of a problem to the beginning to infer a sequence of optimal actions. Extensive form games may have several Nash equilibria and backward induction helps us to pick out a good equilibrium. Any Nash equilibrium found using backward induction is also a Nash equilibrium for every subgame, or equivalently a Subgame Perfect Equilibrium [50].

### 3.1. Game I: OP and Sinks

In the first stage, hereinafter *OP pricing stage*, the OP chooses the price *p* in order to maximize its profit. The optimal price $p^{*}$ is given by the problem

(8) $$p^{*}=\underset{p}{\mathrm{argmax}}\Pi_{OP}\left(p,X_{s}\right).$$

In the second stage, called *WSN subscription game*, sinks decide to subscribe or not to the OP connectivity service based on the perceived utility. Sinks have limited information due to the restrictions in power, processing capabilities and memory [46] and their subscription decisions may not be optimal for their IoT-SP.

#### 3.1.1. WSN Subscription Game

This stage is played once the OP has fixed its price *p*. Sinks equilibrium is solved using the unified framework provided by Population Games described in [48]. This framework is useful for study strategic interactions between agents with certain properties that our model satisfy. Furthermore, the analysis is easily extensible from static to dynamic games, which will allow us to obtain more realistic conclusions in future studies. The equilibrium reached is a Nash equilibrium.

##### Population Game

- *Strategies:* S={0,1}, where 0 means not to subscribe to the OP and 1 means to subscribe to the OP.
- *Social State:* $X_{s}=\left\{x_{0},x_{1}\right\},x_{0}+x_{1}=1$. Sinks distribution between not being served and OP.
- *Payoffs:* $F_{s}\left(x_{0},x_{1}\right)=\left\{F_{s0}\left(X\right),F_{s1}\left(X\right)\right\}=\left\{0,U_{s}\left(3\right)\right\}$, where $F_{s_{0}}\left(X\right)$ is the utility of the users choosing the strategy of not to subscribe to the OP and $F_{s1}\left(X\right)$ is the utility of the users choosing the strategy of subscribe to the OP.

##### Pure Best Response

The first step for solve the population game is to obtain the pure strategies that are optimal at each social state $X_{s}$.

(9) $$b\left(X_{s}\right)\equiv \underset{i\in S}{\mathrm{argmax}}F_{si}\left(X_{s}\right)=\left\{\begin{array}{c} i=1\;\mathrm{if}\;F_{s1}\left(X_{s}\right)\geq F_{s0}\left(X_{s}\right)⟺x_{1}\leq \frac{c-p}{c\tau Nr}\\ i=0\;\mathrm{if}\;F_{s0}\left(X_{s}\right)\geq F_{s1}\left(X_{s}\right)⟺x_{1}\geq \frac{c-p}{c\tau Nr} \end{array}\right..$$

##### Mixed Best Response

Once we have obtained the pure best responses, we can extend the results to include the optimal mixed strategies.

(10) $$B\left(X_{s}\right)\equiv \left\{\left[z_{0}+z_{1}=1;z_{i}\in R_{+}\right]:z_{i}>0\Rightarrow i\in b\left(X_{s}\right)\right\}=\left\{\begin{array}{c} z_{0}=0,z_{1}=1\;\mathrm{if}\;x_{1}\leq \frac{c-p}{c\tau Nr}\\ z_{0}>0,z_{1}>0\;\mathrm{if}\;x_{1}=\frac{c-p}{c\tau Nr}\\ z_{0}=1,z_{1}=0\;\mathrm{if}\;x_{1}\geq \frac{c-p}{c\tau Nr} \end{array}\right..$$

##### Nash Equilibrium

At this point social state $x\in X_{s}$ is a Nash equilibrium of the game $F_{s}$ if all the agents chooses a best response to $x\in X_{s}$:

(11) $$NE\left(F_{s}\right)\equiv \left\{x\in X_{s}:x\in B\left(X_{s}\right)\right\}=\left\{\begin{array}{ccc} \left(0,1\right) & \mathrm{if} & p\leq c\left(1-\tau Nr\right)\\ \left(1-\frac{c-p}{c\tau Nr},\frac{c-p}{c\tau Nr}\right) & \mathrm{if} & c\left(1-\tau Nr\right)\leq p\leq c\\ \left(1,0\right) & \mathrm{if} & p\geq c \end{array}\right..$$

#### 3.1.2. OP Pricing Stage

In this stage, the OP wants to maximize its profit given by Equation (4). Given the three cases obtained from (11) we analyze the case where the maximum profit is reached.

(12) $$\Pi_{OP}=\left\{\begin{array}{ccc} pNr & \mathrm{if} & 0<p\leq c\left(1-\tau Nr\right)\\ p\frac{c-p}{c\tau } & \mathrm{if} & c\left(1-\tau Nr\right)\leq p\leq c\\ 0 & \mathrm{if} & p\geq c \end{array}\right.$$

- Case $0<p\leq c\left(1-\tau Nr\right)$:
  In this case, the maximum profit is obtained solving the optimization problem
  (13) $$\begin{array}{cc} \max_{p} & \Pi_{OP_{1}}^{*}=pNr\\ \mathrm{subject}\;\mathrm{to} & 0<p\leq c\left(1-\tau Nr\right). \end{array}$$
  The solution for the previous problem is
  (14) $$\begin{array}{c} \Pi_{OP_{1}}^{*}=c\left(1-\tau Nr\right)Nr\;\mathrm{if}\;0\leq \tau <\frac{1}{Nr}\;\mathrm{with}\;p^{*}=c\left(1-\tau Nr\right). \end{array}$$
  Note that if τNr>1 the upper limit $c\left(1-\tau Nr\right)$ is negative and, therefore, there is not possible solution for $p^{*}$ in this case.
- Case $c\left(1-\tau Nr\right)\leq p\leq c$:
  In this case, the maximum profit is obtained solving the optimization problem
  (15) $$\begin{array}{cc} \max_{p} & \Pi_{OP_{2}}^{*}=\left(p\frac{c-p}{c\tau }\right)\\ \mathrm{subject}\;\mathrm{to} & c\left(1-\tau Nr\right)\leq p\leq c \end{array}$$
  The problem in (15) is solved using Karush-Kuhn-Tucker (KKT) conditions and its solution is:
  (16) $$\Pi_{OP_{2}}^{*}=\left\{\begin{array}{ccc} c\left(1-\tau Nr\right)Nr & \mathrm{if} & 0\leq \tau <\frac{1}{2Nr}\\ \frac{c}{4\tau } & \mathrm{if} & \tau \geq \frac{1}{2Nr} \end{array}\right..$$
- Case p≥c:
  In this case, for any value of *p* the maximum profit is
  (17) $$\begin{array}{c} \Pi_{OP_{3}}=0. \end{array}$$

Combining (14) and (16) the OP optimal profit can be summarized as:

(18) $$\Pi_{OP}^{*}=\left\{\begin{array}{ccccc} c\left(1-\tau Nr\right)Nr & \mathrm{if} & \tau <\frac{1}{2Nr} & \mathrm{with} & p^{*}=c\left(1-\tau Nr\right)\\ \max\left(\{cNr\left(1-\tau Nr\right),\frac{c}{4\tau }\}\right) & \mathrm{if} & \frac{1}{2Nr}\leq \tau \leq \frac{1}{Nr} & \mathrm{with} & p^{*}=\left[c\left(1-\tau Nr\right),\frac{c}{2}\right]\\ \frac{c}{4\tau } & \mathrm{if} & \frac{1}{Nr}<\tau & \mathrm{with} & p^{*}=\frac{c}{2} \end{array}\right..$$

The expression for the profit in (18) can be simplified given that $\frac{c}{4\tau }\geq cNr\left(1-\tau Nr\right)$ for any value of *c*, *N*, *r* and τ. To prove this we analyze the expressions for any value of τ

(19) $$\begin{array}{c} \frac{c}{4\tau }\geq cNr\left(1-\tau Nr\right), \end{array}$$

re-writing with A=τNr, the previous expression is simplified to:

$$\begin{array}{c} 1\geq 4A-4A^{2}. \end{array}$$

**Figure 4:**
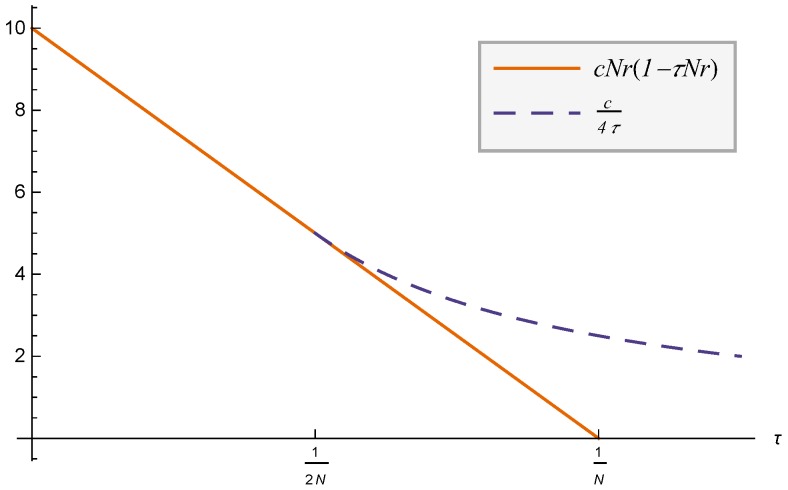
Normalized OP profit (N=1) for each case with c=1, r=1.

We can demonstrate that

(20) $$\max_{A}\left(4A-4A^{2}\right)\leq 1$$

$$\begin{array}{c} \frac{\partial \left(4A-4A^{2}\right)}{\partial A}=0\\ \frac{\partial \left(4A-4A^{2}\right)}{\partial A}=4-8A\\ 4-8A^{*}=0\\ A^{*}=\frac{1}{2}\\ 4A^{*}-4{A^{*}}^{2}=1, \end{array}$$

which proves (19). Figure 4 shows a particular case of the demonstration, where we can see how $\frac{c}{4\tau }\geq cNr\left(1-\tau Nr\right)$ for the range of interest $\tau \geq \frac{1}{2Nr}$. With the previous demonstration, OP optimal profit can be simplified to:

(21) $$\Pi_{OP}^{*}=\left\{\begin{array}{ccc} c\left(1-\tau Nr\right)Nr & \mathrm{if} & \tau <\frac{1}{2Nr}\\ \frac{c}{4\tau } & \mathrm{if} & \frac{1}{2Nr}\leq \tau \end{array}\right..$$

(22) $$p^{*}=\left\{\begin{array}{ccc} c\left(1-\tau Nr\right) & \mathrm{if} & \tau <\frac{1}{2Nr}\\ \frac{c}{2} & \mathrm{if} & \frac{1}{2Nr}\leq \tau \end{array}\right..$$

(23) $$x_{1}^{*}=\left\{\begin{array}{ccc} 1 & \mathrm{if} & \tau <\frac{1}{2Nr}\\ \frac{1}{2\tau Nr} & \mathrm{if} & \frac{1}{2Nr}\leq \tau \end{array}\right..$$

In order to understand better the behavior of the first game we can re-write the equations in terms of the maximum amount of data generated by sensors normalized by the system capacity, which we define as maximum system load *L*:

$$\begin{array}{c} L=\tau Nr=\frac{Nr}{\mu } \end{array}$$

obtaining

(24) $$\Pi_{OP}^{*}=\left\{\begin{array}{ccc} c\left(1-L\right)L\mu & \mathrm{if} & L<\frac{1}{2}\\ \frac{c\mu }{4} & \mathrm{if} & L\geq \frac{1}{2} \end{array}\right..$$

(25) $$p^{*}=\left\{\begin{array}{ccc} c\left(1-L\right) & \mathrm{if} & L<\frac{1}{2}\\ \frac{c}{2} & \mathrm{if} & L\geq \frac{1}{2} \end{array}\right..$$

(26) $$x_{1}^{*}=\left\{\begin{array}{ccc} 1 & \mathrm{if} & L<\frac{1}{2}\\ \frac{1}{2L} & \mathrm{if} & L\geq \frac{1}{2} \end{array}\right..$$

### 3.2. Game II: Internet of Things-Service Providers (IoT-SPs) and Users

The scenario analyzed in this section is a model with two IoT-SPs and M users. In the first stage, also known as *IoT-SPs Pricing stage*, the IoT-SPs compete with the pricing strategies in order to maximize their profits given by (7). This game is solved assuming the solution for Game I obtained above.

#### 3.2.1. Users Subscription Game

This stage is played when the IoT-SPs have decided its prices $f_{j}^{*}$. The concept of equilibrium used for users is Nash equilibrium.

The utility of the users described in (5) is a Logit discrete choice model. In such a model, if the number of users *M* is large enough, it can be proved that the portion of user choosing the IoT-SP${}_{j}$ equals the probability of a user choosing that option [41,51]:

(27) $$P_{j}=\frac{{\left(\frac{R_{j}}{f_{j}}\right)}^{\alpha }}{\sum_{k=0}^{n}{\left(\frac{R_{k}}{f_{k}}\right)}^{\alpha }}=y_{j},$$

where *n* is the number of IoT-SPs and α is the sensitivity parameter described in (5). Given that the utility of the users that do not subscribe is zero $U_{u_{i0}}=0$, the “no-operator” option is characterized by the ratio $\left(\frac{R_{0}}{f_{0}}\right)=1$. The distribution of users choosing each strategy can be expressed as:

(28) $$\begin{array}{c} y_{0}=\frac{1}{{\left(\frac{R_{1}}{f_{1}}\right)}^{\alpha }+{\left(\frac{R_{2}}{f_{2}}\right)}^{\alpha }+1},\\ y_{1}=\frac{{\left(\frac{R_{1}}{f_{1}}\right)}^{\alpha }}{{\left(\frac{R_{1}}{f_{1}}\right)}^{\alpha }+{\left(\frac{R_{2}}{f_{2}}\right)}^{\alpha }+1},\\ y_{2}=\frac{{\left(\frac{R_{2}}{f_{2}}\right)}^{\alpha }}{{\left(\frac{R_{1}}{f_{1}}\right)}^{\alpha }+{\left(\frac{R_{2}}{f_{2}}\right)}^{\alpha }+1}. \end{array}$$

where $y_{0}$ is the fraction of users not subscribed and $y_{1},y_{2}$ are the portion of users subscribed to IoT-SP${}_{1}$ and IoT-SP${}_{2}$ respectively.

#### 3.2.2. IoT-SPs Pricing Stage

In this stage, each IoT-SP want to maximize its own profit given by (7). Given the solution of the previous stage (28) and the solution for Game I in (21) the providers’ profits in the Nash equilibrium are going to be analyzed.

With the solution of OP-Sinks game and users subscription game we can re-write the profit for the IoT-SP${}_{i}$ as:

(29) $$\begin{array}{c} \Pi_{{\mathrm{IoT-SP}}_{i}}\left(f_{1},f_{2}\right)=\frac{f_{i}M{\left(\frac{R_{i}}{f_{i}}\right)}^{\alpha }}{{\left(\frac{R_{1}}{f_{1}}\right)}^{\alpha }+{\left(\frac{R_{2}}{f_{2}}\right)}^{\alpha }+1}-pR_{i},\;i=1,2. \end{array}$$

In order to find the Nash equilibrium we use the best response functions for both operators defined as follows:

$$\begin{array}{c} BR_{1}\left(f_{2}\right)=f_{1}^{*}\left(f_{2}\right)=\arg\max_{f_{1}\;>\;0}\Pi_{{\mathrm{IoT-SP}}_{1}}\left(f_{1},f_{2}\right),\\ BR_{2}\left(f_{1}\right)=f_{2}^{*}\left(f_{1}\right)=\arg\max_{f_{2}\;>\;0}\Pi_{{\mathrm{IoT-SP}}_{2}}\left(f_{1},f_{2}\right). \end{array}$$

The Nash equilibrium is obtained from the equation system

(30) $$\begin{array}{c} f_{1}^{*}={\mathrm{argmax}}_{f_{1}}\Pi_{{\mathrm{IoT-SP}}_{1}}\left(f_{1},f_{2}^{*}\right)\;s.t.\;f_{1}>0\\ f_{2}^{*}={\mathrm{argmax}}_{f_{2}}\Pi_{{\mathrm{IoT-SP}}_{1}}\left(f_{1}^{*},f_{2}\right)\;s.t.\;f_{2}>0. \end{array}$$

In order to obtain the optimum prices we equal the partial derivatives to zero

$$\begin{array}{cc} \frac{\partial \Pi_{{\mathrm{IoT-SP}}_{1}}\left(f_{1},f_{2}\right)}{\partial f_{1}} & =\frac{M{\left(\frac{R_{1}}{f_{1}}\right)}^{\alpha }\left(-\alpha +{\left(\frac{R_{1}}{f_{1}}\right)}^{\alpha }-\left(\alpha -1\right){\left(\frac{R_{2}}{f_{2}}\right)}^{\alpha }+1\right)}{{\left({\left(\frac{R_{1}}{f_{1}}\right)}^{\alpha }+{\left(\frac{R_{2}}{f_{2}}\right)}^{\alpha }+1\right)}^{2}}=0,\\ \frac{\partial \Pi_{{\mathrm{IoT-SP}}_{2}}\left(f_{1},f_{2}\right)}{\partial f_{2}} & =\frac{M{\left(\frac{R_{2}}{f_{2}}\right)}^{\alpha }\left({\left(\frac{R_{2}}{f_{2}}\right)}^{\alpha }-\left(\alpha -1\right)\left({\left(\frac{R_{1}}{f_{1}}\right)}^{\alpha }+1\right)\right)}{{\left({\left(\frac{R_{1}}{f_{1}}\right)}^{\alpha }+{\left(\frac{R_{2}}{f_{2}}\right)}^{\alpha }+1\right)}^{2}}=0. \end{array}$$

With the change $Ai={\left(\frac{R_{i}}{f_{i}}\right)}^{\alpha }$ and simplifying the system we obtain

(31) $$\begin{array}{c} A1=(\alpha -1)(A2+1), \end{array}$$

(32) $$\begin{array}{c} A2=(\alpha -1)(A1+1). \end{array}$$

Solving the previous equation system we obtain

(33) $$\begin{array}{c} A1^{*}=A2^{*}=\frac{1-\alpha }{\alpha -2}. \end{array}$$

**Figure 5:**
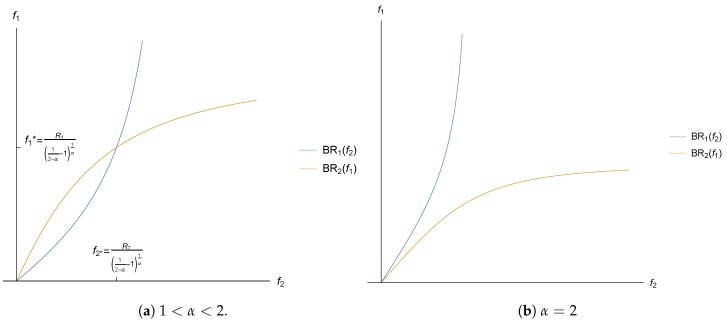
Best responses with different values of α.

Given that $R_{i}$ and $f_{i}$ are positive, Ai has to be positive. From Equation (33) we can infer that 1<α≤2, otherwise Ai would be negative, and there would be no real solutions for $f_{i}$. In addition, we see that there is only one pricing equilibrium different than $\left(f_{1}^{*}=0,f_{2}^{*}=0\right)$ if and only if 1<α<2. Figure 5a shows a particular solution when 1<α<2, where we observe that there is a Nash equilibrium where the best response functions intersect. On the other hand, Figure 5b shows how the best response functions of both operators only intersect in (0,0), and therefore the only possible solution when α=2 is $\left(f_{1}^{*}=0,f_{2}^{*}=0\right)$.

Reverting the change $f_{i}=R_{i}{\mathrm{Ai}}^{-\frac{1}{\alpha }}$, we get the pricing strategies for both IoT-SPs in the equilibrium

(34) $$\begin{array}{c} f_{1}^{*}={\left(\frac{1}{2-\alpha }-1\right)}^{-1/\alpha }R_{1}\;s.t.\;1<\alpha <2,\\ f_{2}^{*}={\left(\frac{1}{2-\alpha }-1\right)}^{-1/\alpha }R_{2}\;s.t.\;1<\alpha <2. \end{array}$$

Replacing (34) in (28) we obtain the users distribution in the equilibrium, that depends only on α.

(35) $$\begin{array}{c} y_{0}^{*}=\frac{2}{\alpha }-1,\;y_{1}^{*}=\frac{\alpha -1}{\alpha },\;y_{2}^{*}=\frac{\alpha -1}{\alpha }. \end{array}$$

**Figure 6:**
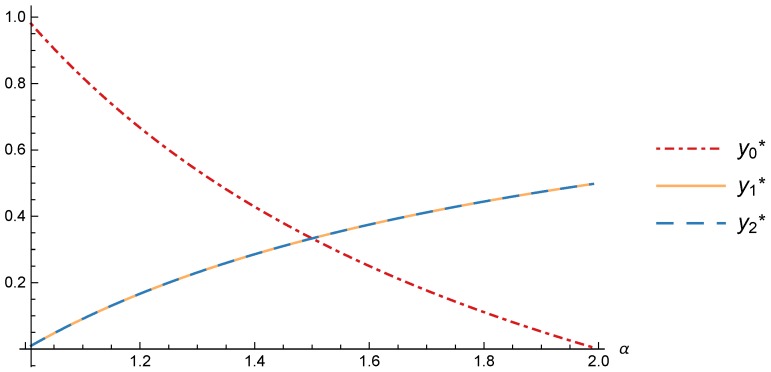
Distribution of the users between the strategies in the equilibrium.

Figure 6 shows how when the value of α is close to 1 the percentage of users that subscribe is very small and the prices of the providers are very high. This could be counter-intuitive, but it explains cases where some users are willing to pay huge amounts of money even without clear evidence of a good quality of service. On the other hand, when α is close to 2, almost all the users decide to subscribe. This is caused because all the users act in a more rational behavior, and the providers adjust its prices in order to attract the largest possible number of them.

Finally, replacing the values obtained in (22), (23) and (34) in (29) we obtain the profits in the equilibrium for both operators

(36) $$\Pi_{{\mathrm{IoT-SP}}_{1}}^{*}=\left\{\begin{array}{ccc} N_{1}r\left(c\left(\tau Nr-1\right)+\frac{\left(\alpha -1\right){\left(\frac{1}{2-\alpha }-1\right)}^{-1/\alpha }M}{\alpha }\right) & \mathrm{if} & \tau <\frac{1}{2Nr}\\ \frac{N_{1}}{4N\tau }\left(\frac{2{\left(\frac{1}{2-\alpha }-1\right)}^{-1/\alpha }\left(\alpha -1\right)M}{\alpha }-c\right) & \mathrm{if} & \tau \geq \frac{1}{2Nr} \end{array}\right.$$

(37) $$\Pi_{{\mathrm{IoT-SP}}_{2}}^{*}=\left\{\begin{array}{ccc} N_{2}r\left(c\left(\tau Nr-1\right)+\frac{\left(\alpha -1\right){\left(\frac{1}{2-\alpha }-1\right)}^{-1/\alpha }M}{\alpha }\right) & \mathrm{if} & \tau <\frac{1}{2Nr}\\ \frac{N_{2}}{4N\tau }\left(\frac{2{\left(\frac{1}{2-\alpha }-1\right)}^{-1/\alpha }\left(\alpha -1\right)M}{\alpha }-c\right) & \mathrm{if} & \tau \geq \frac{1}{2Nr} \end{array}\right.$$

Analyzing the previous results we observe that $\Pi_{{\mathrm{IoT-SP}}_{i}}^{*}>0$ if the following conditions are met:

Case $\tau <\frac{1}{2Nr}$:

(38) $$\begin{array}{c} M>\frac{{\left(\frac{1}{2-\alpha }-1\right)}^{1/\alpha }\alpha c\left(1-\tau Nr\right)}{\alpha -1}. \end{array}$$

Case $\tau \geq \frac{1}{2Nr}$:

(39) $$\begin{array}{c} M>\frac{{\left(\frac{1}{2-\alpha }-1\right)}^{1/\alpha }\alpha c}{2(\alpha -1)}. \end{array}$$

**Figure 7:**
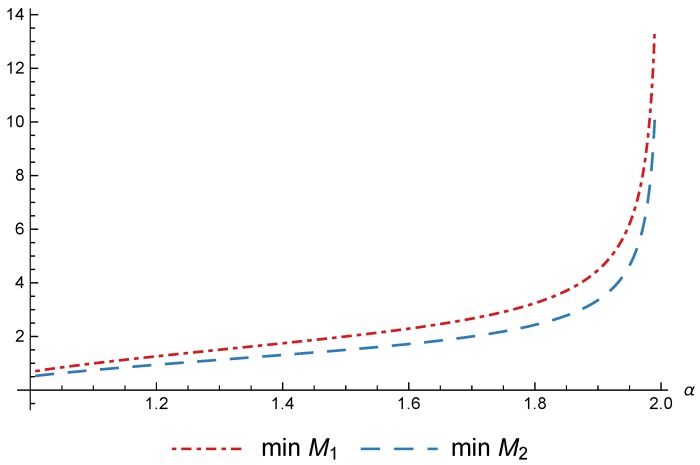
Minimum value of *M* to obtain positive profits with c=1 and τNr=1/3.

Restrictions (38) and (39)are represented in Figure 7 as $minM_{1}$ and $minM_{2}$ respectively. When the value of α is near to 1, the impact of the ratio R/f in users’ utility is low, and the providers can increase their prices, obtaining, as shown, positive profits with a very small pool of users. However, when the value of α increases the providers have to decrease its prices in order to attract users and the revenue per user decreases drastically, while the cost of sensor data collection remains constant. In order to obtain positive profits an increasing number of users *M* is needed as α increases, with an asymptotic behavior in α=2.

## 4. Results and Discussion

In this section, we present the numerical results for the games analyzed in the previous section. The results were obtained for the reference case shown in Table 1 unless otherwise specified. The figures are structured as follows: Figure 8, Figure 9 and Figure 10 are related to Game I, while Figure 11, Figure 12, Figure 13, Figure 14, Figure 15 and Figure 16 are related to Game II-IoT-SP${}_{1}$ and Figure 17, Figure 18, Figure 19, Figure 20, Figure 21 and Figure 22 are related to Game II-IoT-SP${}_{2}$.

### 4.1. OP Pricing and Profit

In order to study the Game I results we show the optimal price $p^{*}$ and the OP profit $\Pi_{OP}$, varying the maximum system load *L* and the parameter *c*.

**Figure 8:**
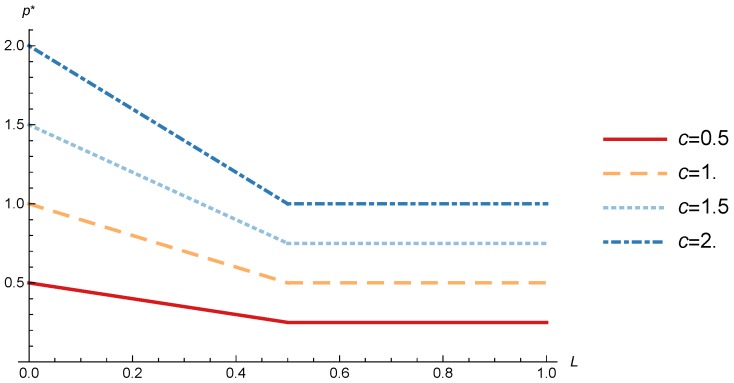
OP optimal price as a function of *L* for different values of *c*.

**Figure 9:**
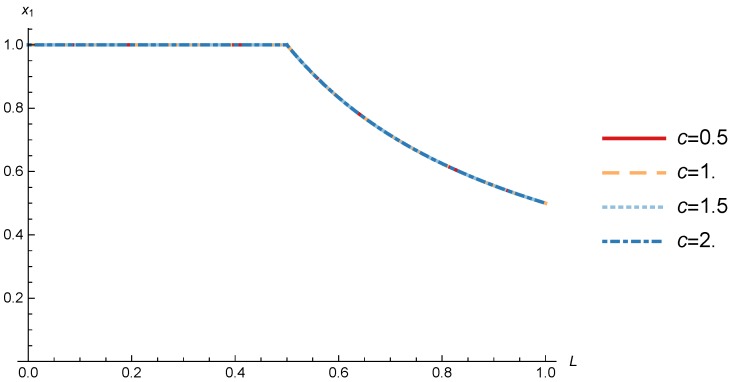
Social state as a function of *L* for different values of *c*.

Figure 8 shows the OP optimal price as a function of *L* for different values of *c*. When *c* increases, the optimal price increases as expected, given that *c* acts as a conversion factor. More interesting is the behavior of the price when it is analyzed in terms of the maximum system load *L*. When the maximum system load (eq. L) increases the utility of the sinks decreases given the growing mean transmission time. When $L<\frac{1}{2}$ the OP decreases its price and thanks to it all the sinks decide to subscribe. Nevertheless, when the generated traffic is more than the half of the network capacity is more profitable for the OP to keep constant the price and decrease the percentage of subscribed sinks as shown in Figure 8 and Figure 9. In terms of real system load $L_{R}=x_{1}L$, it is equivalent to the maximum system load while L≤1/2, but when L>1/2 the real load remains constant in $L_{R}=1/2$, which means that real system load never exceeds the 50% of the capacity. Another approximation studied in [33] where different priorities were used in the OP wireless network obtained a better efficiency. In order to implement this improvement in our model a sensing data differentiation in delay requirements is needed, where priority traffic has a more restrictive utility function, while non-priority traffic utility function is more relaxed. This would allow us to obtain a better efficiency in the OP network and, in addition, allows the IoT-SPs to offer new services using the sensing data with lower requirements.

**Figure 10:**
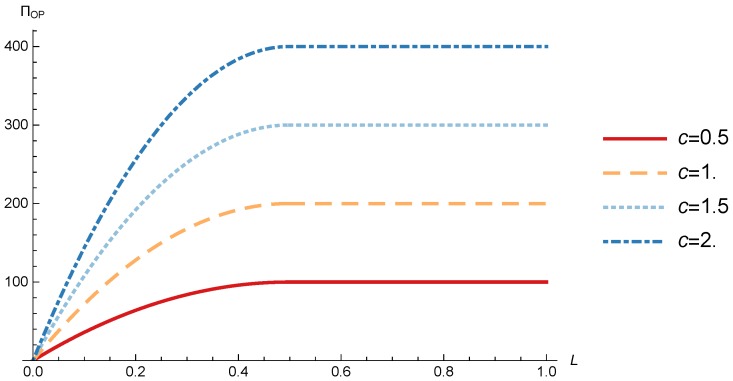
OP optimal profit as a function of *L* for different values of *c*.

Figure 10 shows the OP profit as a function of *L* for different values of *c*. The figure shows how the OP profit increases when the system load increases until $L=\frac{1}{2}$. After this point, the profit remains constant with the system load. In addition OP profit also experiments an increase with *c* for any value of *L*.

### 4.2. IoT-SP${}_{1}$ and IoT-SP${}_{2}$ Pricing and Profits

In order to study the Game II results we show the equilibrium price $f_{1}^{*}$ and the IoT-SP${}_{1}$ profit $\Pi_{IoT-SP_{1}}$, varying the sensitivity of the users to the providers’ price α and the parameters *c*, *N* and *M*.

**Figure 11:**
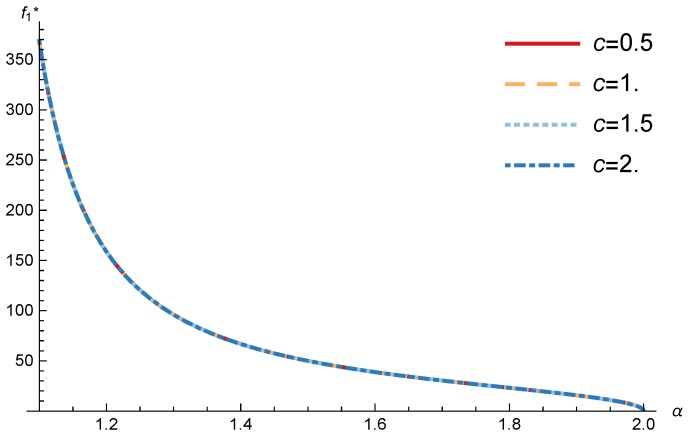
IoT-SP${}_{1}$ equilibrium price as a function of α for different values of *c*.

**Figure 12:**
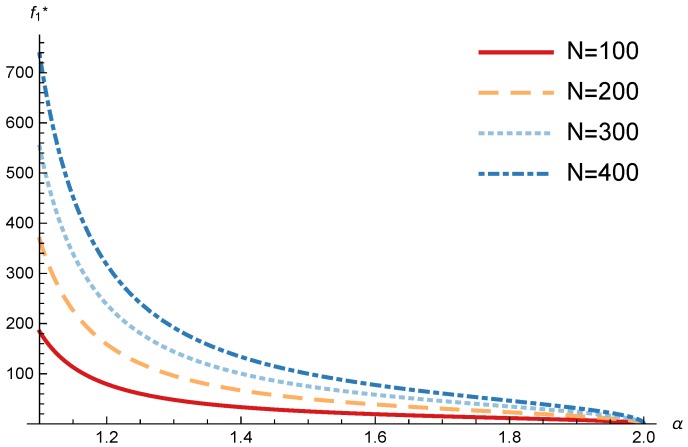
IoT-SP${}_{1}$ equilibrium price as a function of α for different values of *N*.

Figure 11 and Figure 12 show the IoT-SP${}_{1}$ equilibrium price $f_{1}^{*}$ as a function of α for different values of *c* and *N* respectively. The equilibrium price does not depend on the value of *c* and increases with N, due to the higher utility perceived by users. Note that it only happens if the maximum system load L<1/2. We also observe that when users’ sensitivity to $R_{1}/f_{1}$ increases, the IoT-SP${}_{1}$ optimal price decreases very fast.

**Figure 13:**
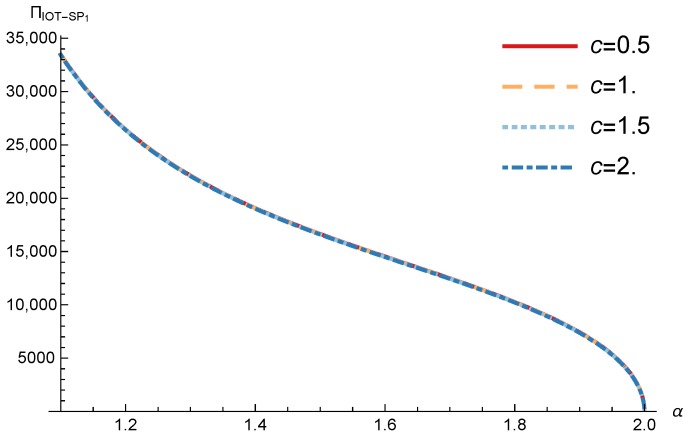
IoT-SP${}_{1}$ equilibrium profit as a function of α for different values of *c*.

**Figure 14:**
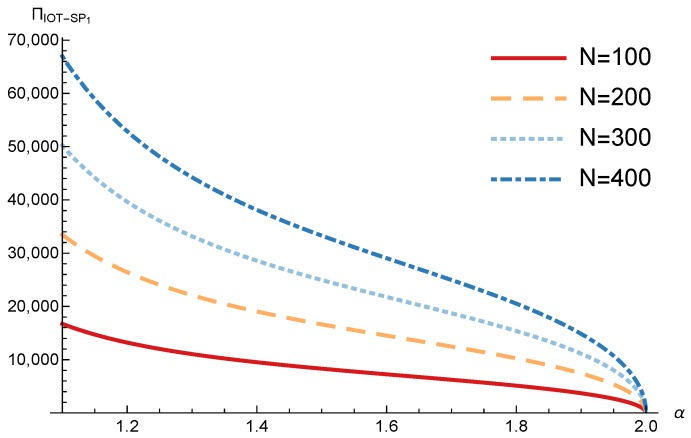
IoT-SP${}_{1}$ equilibrium profit as a function of α for different values of *N*.

**Figure 15:**
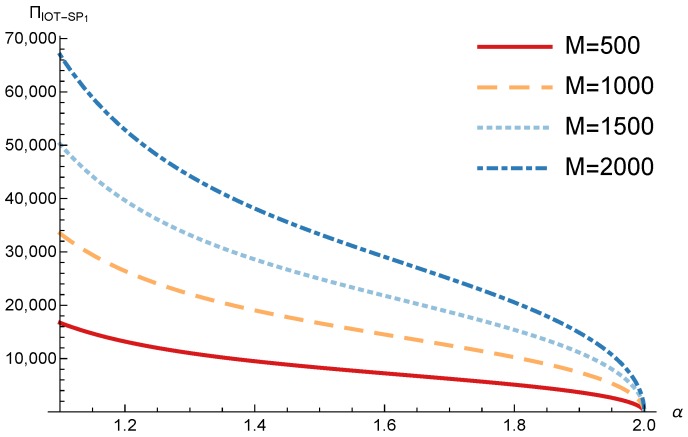
IoT-SP${}_{1}$ equilibrium profit as a function of α for different values of *M*.

Figure 13 shows the IoT-SP${}_{1}$ profit as a function of α for different values of *c*. Similarly to the price the equilibrium profit does not depend on the value of *c* and decreases with the sensitivity of the users to the price. On the other hand Figure 14 shows that the IoT-SP${}_{1}$ profit increases with the value of N. This means that final users are willing to pay a higher price if the amount of data collected by the IoT-SP is higher. This will drive to a competition between the IoT-SPs to increase the number of sensors that is not studied in this paper. As shown in Figure 15 the profit also increases with M due to the higher pool of users subscribed to the IoT-SP${}_{1}$. Note that here there is not a congestion effect when M increases. With low values of the sensitivity parameter users choices have a very weak dependence on the prices $f_{i}$ and the IoT-SPs increase hugely its prices. When it occurs the rate of users subscribed to the IoT-SPs is very low, but the higher prices offset it.

**Figure 16:**
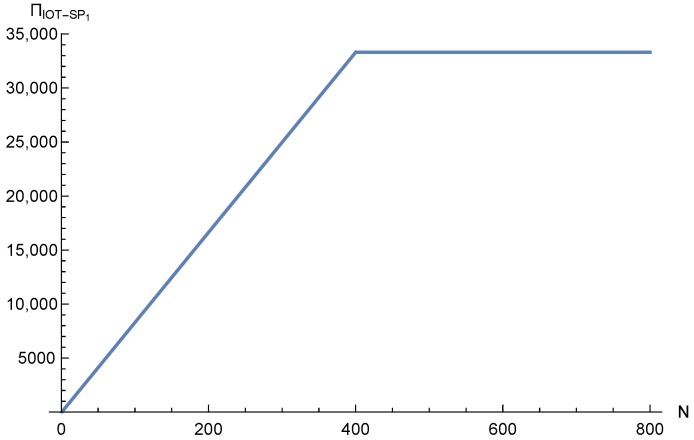
IoT-SP${}_{1}$ equilibrium profit as a function of *N*.

Figure 16 shows the IoT-SP${}_{1}$ profit as a function of *N*. The figure shows how the profit increases with N until the OP network is congested. After that, the profit remains constant.

**Figure 17:**
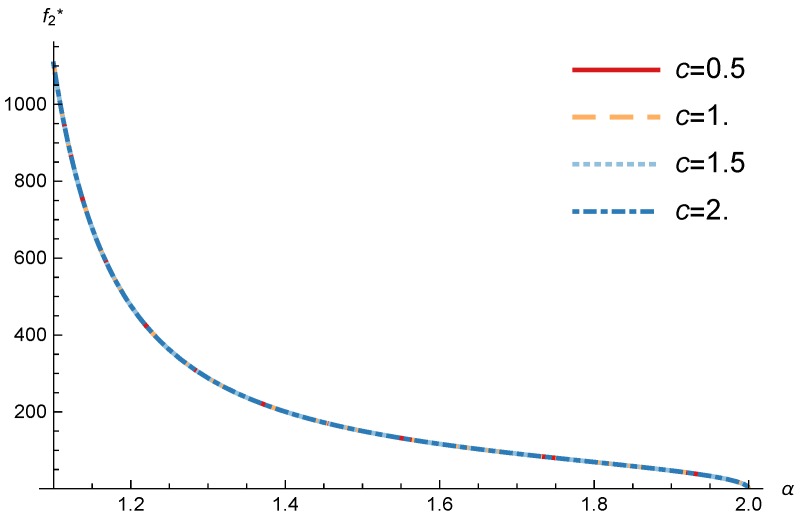
IoT-SP${}_{2}$ equilibrium price as a function of α for different values of *c*.

**Figure 18:**
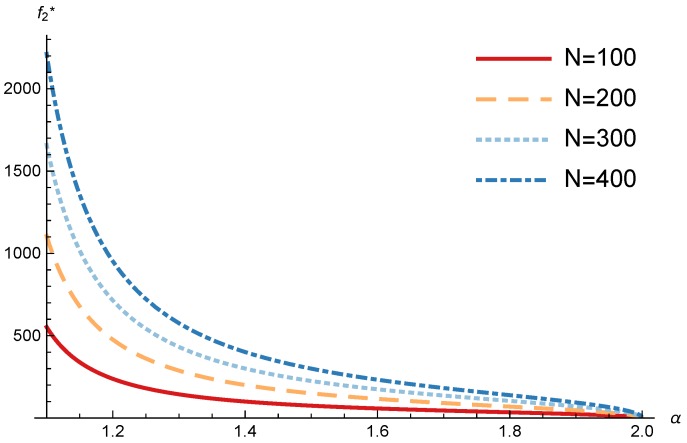
IoT-SP${}_{2}$ equilibrium price as a function of α for different values of *N*.

**Figure 19:**
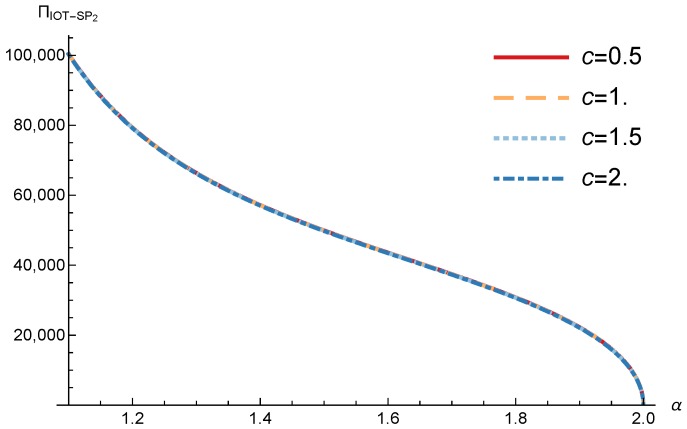
IoT-SP${}_{2}$ equilibrium profit as a function of α for different values of *c*.

**Figure 20:**
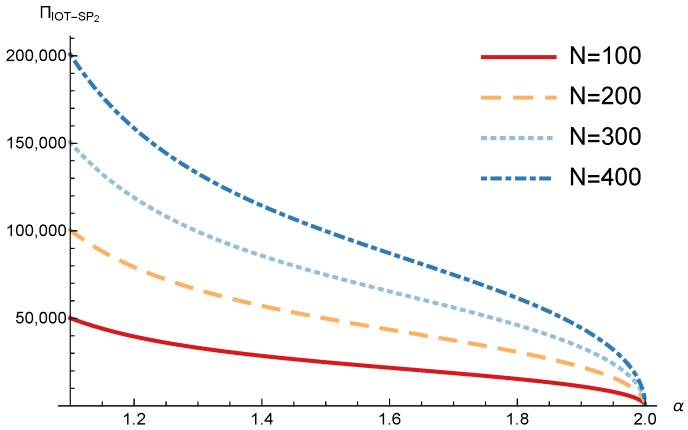
IoT-SP${}_{2}$ equilibrium profit as a function of α for different values of *N*.

**Figure 21:**
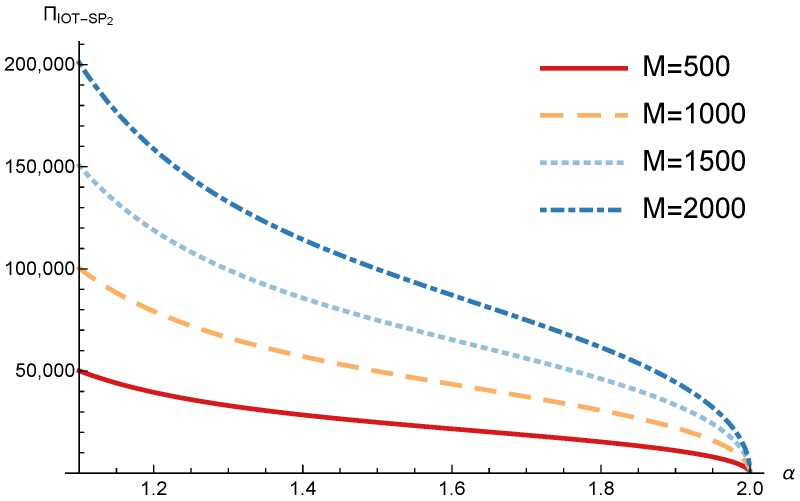
IoT-SP${}_{2}$ equilibrium profit as a function of α for different values of *M*.

**Figure 22:**
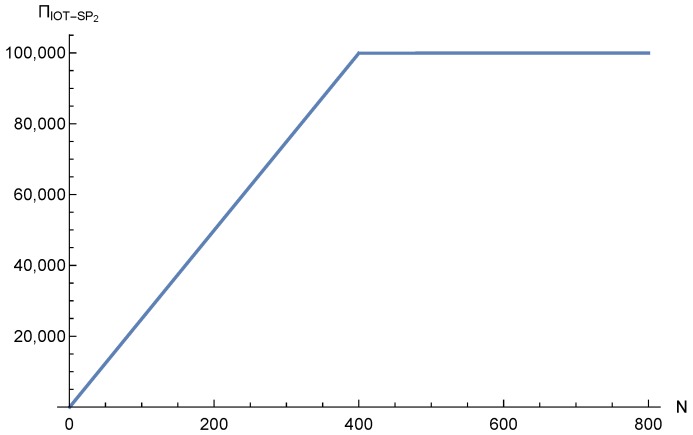
IoT-SP${}_{2}$ equilibrium profit as a function of *N*.

The conclusions for the IoT-SP${}_{2}$ obtained from Figure 17, Figure 18, Figure 19, Figure 20, Figure 21 and Figure 22 are the same that those obtained for the IoT-SP${}_{1}$ taking into account that the values of N1 and N2 are different.

From previous results we observe that users’ sensitivity parameter α is critical in the second game. For values of α<1 and α>2 it is not possible to reach an equilibrium with positive profits, as deduced from the analysis. In addition, if the value is in the range 1<α<2 it still has a huge relevance in the IoT-SPs equilibrium decisions and profits, not only in the value of them, but also in the feasibility of the whole model as deduced in (38) and (39).

## 5. Conclusions

A novel network model for providing IoT-based services with private sensor networks, using third party access infrastructure, has been studied. The model was analyzed as two games using game theory, population games, Logit discrete choice model, optimization and Nash equilibrium concepts.

Firstly, a congestion model was proposed for the utility of the sensors and it was shown economically viable for the network operator to offer connectivity service, however, the system load never exceeded half of system the capacity.

Secondly, a Logit discrete choice model was chosen to model users’ decisions with two Internet of Things Service Providers competing for serving them maximizing their own profits. It has been shown that, in the equilibrium, both IoT-SPs obtain the same profits multiplied by the portion of the total sensors that each one has. We observed that the value of users’ sensitivity to the data quality/price had to be 1<α<2, in order to obtain a providers’ pricing equilibrium other than (0,0). In addition, the number of potential clients had to be high enough to guarantee the feasibility for the IoT-Service Providers.

Given that both stages have been shown feasible under specific conditions, we can conclude that the whole network model is conditionally feasible from an economic point of view.

Future work will involve a sensor-data traffic differentiation model, with different delay requirements and different pricing schemes for each sensor-data traffic type, in order to improve the efficiency on the operator wireless network and create new business opportunities for the providers.

## Figures and Tables

**Table 1:** Reference Case.

Parameter	Value
Quality conversion factor (*c*)	1
Sensor data generation ratio (*r*)	1
Mean sensing-data-unit transmission time (1/μ)	1/800
Total Number of sensors (*N*)	200
Number of IoT-SP${}_{1}$ sensors (N1)	$\frac{1}{4}N$
Number of IoT-SP${}_{2}$ sensors (N2)	$\frac{3}{4}N$
Number of users (*M*)	1000
Sensitivity (α)	1.5
